# Evaluating Executive Functions in Patients with Juvenile Myoclonic Epilepsy Using Frontal Assessment Battery

**DOI:** 10.1155/2020/8710373

**Published:** 2020-09-10

**Authors:** Hossein Sanjari Moghaddam, Masoud Doost Hoseini, Mohammad Reza Khaleghi, Abbas Tafakhori, Mahsa Dolatshahi, Shayan Pourmirbabaei, Elmira Agah, Shakila Meshkat, Vajiheh Aghamollaii

**Affiliations:** ^1^School of Medicine, Tehran University of Medical Sciences, Tehran, Iran; ^2^Neurology Department, Roozbeh Psychiatric Hospital, Tehran University of Medical Sciences, Tehran, Iran; ^3^Students' Scientific Research Center, Tehran University of Medical Sciences, Tehran, Iran; ^4^ImmunoPharmacology Network (IPhoNe), Universal Scientific Education and Research Network (USERN), Tehran, Iran; ^5^Iranian Center of Neurological Research (ICNR), Tehran University of Medical Sciences, Tehran, Iran; ^6^NeuroImmunology Research Association (NIRA), Universal Scientific Education and Research Network (USERN), Tehran, Iran

## Abstract

**Objective:**

In this study, we aimed to evaluate the executive profile of juvenile myoclonic epilepsy (JME) patients using the Frontal Assessment Battery (FAB) as a bedside screening tool and investigate its association with seizure proximity, family history of epilepsy, and polytherapy/monotherapy with antiepileptic drugs (AEDs).

**Background:**

JME patients have deficits in various aspects of executive functions. FAB has proved to be a useful tool for evaluating executive functions in clinical settings.

**Methods:**

Thirty-one JME patients and 110 healthy controls (HCs) were enrolled in this study. The participants were assessed using six subsets of FAB, including conceptualization, mental flexibility, motor programming, sensitivity to interference, inhibitory control, and environmental autonomy.

**Results:**

Compared to HCs, JME patients showed lower scores in conceptualization, mental flexibility, programming, sensitivity to interference, and total FAB. The number of AEDs (polytherapy versus monotherapy) and duration of time since the last seizure had no significant effect on FAB scores in JME patients. We found significant associations between disease duration and conceptualization, mental flexibility, inhibitory control, and total FAB score only in JME patients with recent seizure. Finally, receiver operating characteristic (ROC) analysis showed area under the curve (AUC) of 0.971 (95% confidence interval (CI): 0.947–0.994) for FAB total score, 0.933 for conceptualization (95% CI: 0.973-894), and 0.836 for mental flexibility (95% CI: 0.921-751).

**Conclusions:**

In summary, JME patients had deficits in different aspects of executive functions. FAB is a useful clinical tool for evaluation of executive functions in JME patients.

## 1. Introduction

Juvenile myoclonic epilepsy (JME) is an adolescent-onset idiopathic generalized epilepsy syndrome, which constitutes around 5% to 10% of all epilepsies and 18% of generalized epilepsies [1]. JME is mainly characterized by seizures with repetitive, arrhythmic, and irregular myoclonic jerks, which are predominantly present in the arms after awakening [2]. It may be associated with generalized tonic-clonic seizures (GTCS) or less frequently with absence seizures.

Although patients with JME have average intelligence, JME is usually associated with cognitive impairments in various areas of cognition, including concept formation, abstract reasoning, cognitive speed, planning, and organization [3]. Executive functions are reflected as high-level processes required for a variety of cognitive abilities including attention, the formation of complex thoughts, and performing appropriate behaviors [4] which are mainly dependent on the intact function of frontal lobes. Although the degree of executive dysfunction in JME patients is variable [5], many studies in JME populations have emphasized selective impairment of executive functions in JME [6–8]. Importantly, executive dysfunction has shown an association with more prevalence of psychiatric symptoms and higher frequency of seizures, imposing a high burden on both the patient and the community [6].

Several neuropsychological studies have assessed executive functioning in JME [3, 7, 9, 10]. The standard neuropsychological tests for the assessment of executive functions are time-consuming and require quite experienced administrators. Thus, a screening tool which can detect executive dysfunction in a short time seems to be beneficial. Frontal Assessment Battery (FAB), which was first introduced in 2000 by Dubois et al. [11], is a sensitive test for evaluating executive functioning. FAB consists of six subtests and measures different aspects of frontal lobe function. These six subtests include conceptualization (similarity), mental flexibility (fluency), motor programming (Luria motor series), sensitivity to interference (conflicting instructions), inhibitory control (Go-No-Go task), and environmental autonomy (prehension behavior). It takes about 10 minutes to be completed and is designed to investigate the aspects of executive functions, which are predominantly reliant on the intact integrity and functionality of frontal lobes.

In recent years, FAB has been progressively used as a brief screening test for identifying subtle executive deficits in neurological disorders of frontal and striatal regions, including amyotrophic lateral sclerosis [12, 13], Huntington's disease [14], multiple system atrophy, and progressive supranuclear palsy [15]. The Persian version of FAB was demonstrated to be reliable and valid for assessing the executive functions in Parkinson's disease [16] and was also applied for temporal lobe epilepsy (TLE) [17].

Although FAB is not a comprehensive test for the assessment of executive deficits, it is much easier, cheaper, and shorter to administer, compared to the traditional neuropsychological batteries. Thus, FAB is an optimal test for the assessment of executive deficits in the clinical setting where there is a need for quick and efficient assessment of executive functions in patients with neurological disorders.

In this study, we aimed to evaluate the executive functions in JME patients using FAB. This is the first study, which surveys the utility of FAB in JME patients and its association with clinical and demographic features of JME patients. Some previous studies have hypothesized that seizure proximity, family history of epilepsy, and the number of antiepileptic drugs (AEDs) may affect the cognitive functions [10, 17–19]. To address these hypotheses, we aimed to investigate the applicability of this test in those with and without a recent seizure, those with positive or negative family history, and those receiving monotherapy or polytherapy.

## 2. Materials and Methods

### 2.1. Participants

This is a case-control study, conducted in a group of patients with JME, which were referred from two epilepsy clinics (Yalda clinic and specialized epilepsy clinic of Imam Khomeini Hospital) to Roozbeh Hospital of Tehran University of Medical Sciences (TUMS) for neuropsychological assessment. The eligible patients were selected by simple random sampling from those referred to our center. Healthy controls (HCs) were enrolled using convenience sampling either from family members of JME patients or through online advertisement. An experienced neuropsychiatrist assessed all HCs for neurologic and psychiatric disorders and other exclusion criteria of the study. A total of 110 HCs with no history of neurologic or psychiatric disorders or usage of central nervous system affecting drugs were voluntarily enrolled in our study. Finally, a total of 31 JME patients and 110 HCs were enrolled in this study. Two experienced neurologists made the diagnosis of JME based on clinical features and video-electroencephalography monitoring for confirmation. Also, exclusion criteria were as follows: (1) presence of any concomitant neurologic disease other than epilepsy based on comprehensive history taking and physical examination by an experienced neurologist, (2) having a history of psychiatric diseases based on comprehensive history taking by an experienced psychiatrist, (3) any history of intellectual disability, (4) any history of alcohol or substance abuse, or (5) any other drugs or medical conditions that interfere with cognitive functions. The Wechsler Adult Intelligence Scale (WAIS-111) was used to determine the intelligence quotient of all participants. The patients and HCs were comparable based on intelligence quotient. There was no significant difference between JME patients and HCs regarding sex. Moreover, 45.2% of JME patients and 3.6% of HCs had a family history of seizure, and GTCS was experienced by 83.9% of JME patients. The protocol of this study was approved by the ethical committee of TUMS (the ethics code: IR.TUMS.REC.1395.2470) and is in accordance with The Code of Ethics of the World Medical Association (Declaration of Helsinki). We acquired informed written consents from all of the study participants.

### 2.2. Frontal Assessment Battery

The detailed information on FAB administration and scoring processes are documented in the previous study on the cognitive status of TLE patients [17]. Briefly, a clinical neurologist interviewed all patients, and demographic and seizure-related characteristics were recorded. Subsequently, a trained medical student administered the FAB. FAB has been validated in the Persian language [16]. The six subtests of FAB are:

- *Conceptualization* (*similarity*): participants were asked to determine the category of two or more objects from the same semantic group. For instance, apple, peach, and banana belong to which category?
- *Mental flexibility* (*fluency*): participants were asked to name as many words as they can that begin with the sound “B” except for proper nouns, within the 60 s
- *Motor programming* (*Luria motor series*): participants were first trained how to play the Luria series' fist, edge, and palm, and then, they were asked to do it repetitively by themselves for six times
- *Sensitivity to interference* (*conflicting instructions*): participants were asked to tap on the table twice if the examiner tapped once and to tap once if the examiner tapped twice
- *Inhibitory control* (*Go-No-Go Task*): participants were asked to tap on the table once if the examiner tapped once and not to tap (not to do anything) if the examiner tapped twice
- *Environmental autonomy* (*prehension behavior*): while the participants' hands were placed palms upon their knees, the examiner touched the patients' palms without saying anything. Examiner tried this again and said “Do not take my hands” if the patient had grabbed her hands in the first time

According to the FAB scoring system, the minimum and maximum scores for each task are 0 and 3, respectively. Calculated scores for the six subtests of FAB were summed and documented as the “total FAB score,” similar to our previous study [17].

### 2.3. Statistical Analysis

We used the Statistical Package for the Social Sciences version 23 to execute the statistical analysis of this study. We applied the Shapiro–Wilk test for the assessment of the distribution of study variables. Categorical variables (sex, type of treatment, family history, number of patients with GTCS, and number of patients with and without seizure) were compared by the Chi-square test. Comparison of continuous variables (age, disease duration, FAB total score) was performed using the Student *t*-test. Scores of FAB subsets were compared using one-way analysis of covariance (ANCOVA) controlling for age or age, sex, family history, and disease duration, whenever appropriate. In order to control for type 1 error, the Benjamini-Hochberg correction was performed, and false discovery rate (FDR) was reported. We reported Cohen's *d* for variables with significant results as the indicator for effect size. Partial correlation analysis controlling for age, sex, and family history was performed to investigate the association between disease duration and FAB subset scores, and Pearson's correlation coefficients were reported. For diagnostic efficiency analysis, we performed the receiver operating characteristic (ROC) curve, and the area under the curve (AUC) was reported. FDR and *P* value < 0.05 were considered the cutoff for statistical significance.

## 3. Results

Demographic and clinical characteristics of the study participants are illustrated in Table 1. HCs had better scores in total FAB comparing with JME patients (*P* value < 0.0001).

### 3.1. Between Group Analysis of FAB Scores

#### 3.1.1. FAB Score Differences between JME Patients and HCs

**Figure 1:**
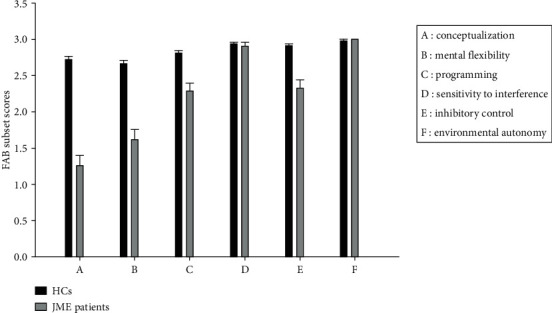
Comparison of FAB subset scores between JME patients and HCs.

Table 2 illustrates the scores of the study participants in six subsets of FAB. In comparison with HCs, JME patients showed worse scores with large effect sizes in conceptualization (FDR < 0.0001 and Cohen′s *d* = 2.26), mental flexibility (FDR < 0.0001 and Cohen′s *d* = 1.60), and programming (FDR < 0.0001 and Cohen′s *d* = 1.03) and lower score with small effect size in interference (FDR < 0.0001 and Cohen′s *d* = 0.11) (Figure 1).

#### 3.1.2. FAB Score Differences between JME Patients with and without a Family History

Approximately 45.2% of JME patients had a family history of epilepsy. After controlling for covariates and multiple comparisons, there was no significant difference between these two groups in five subsets of FABs consisting of conceptualization, mental flexibility, inhibitory control, sensitivity to interference, and environmental autonomy as well as total FAB score (FDR = 0.354, 0.918, 1, 0.4, 1, and 0.597, respectively). Patients with a family history had significantly higher scores in programming (*P* value = 0.014), which did not survive the correction for multiple comparisons (FDR = 0.084).

#### 3.1.3. FAB Score Differences between Patients with and without Recent Seizures

**Figure 2:**
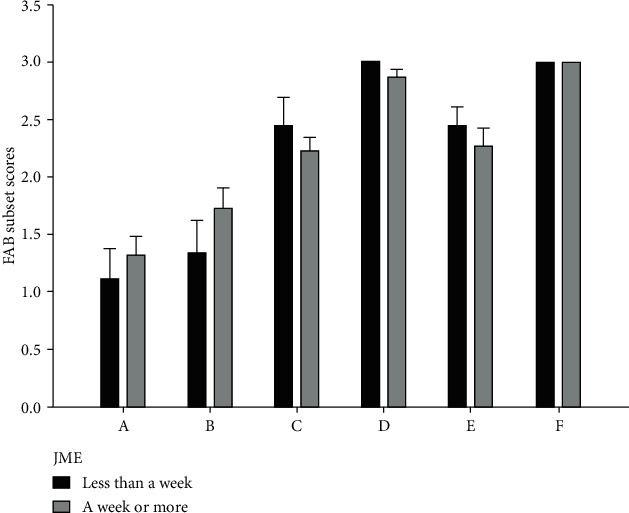
Comparison of FAB subset scores between JME patients with and without recent seizures.

It has been suggested that patients with epilepsy tend to have more severe cognitive impairments in a period after seizures [17]. To investigate this phenomenon in JME patients, we divided the patients into two groups based on the time from the last seizure. Within JME patients, 9 (29%) mentioned the occurrence of a seizure within one week from the time of evaluation, and 22 of them (71%) had experienced a seizure more than one week from the time of evaluation. After controlling for age, sex, family history, disease duration, and multiple comparisons, there were no significant differences between the two groups of JME patients regarding total and FAB subset scores (Table 3 and Figure 2).

#### 3.1.4. FAB Score Differences between Patients on Monotherapy and Polytherapy

**Figure 3:**
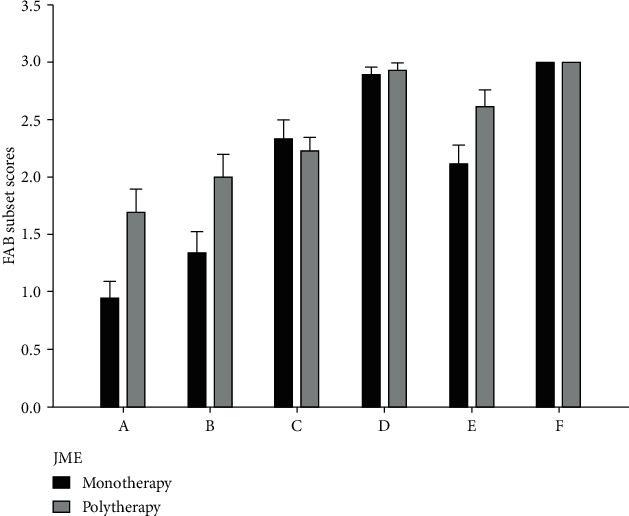
Comparison of FAB subset scores between JME patients on monotherapy and polytherapy regimen.

In order to investigate the effects of AEDs on the performance of patients in FAB, we divided our patients based on their type of treatment into two groups of “monotherapy” and “polytherapy” (Table 4). The results of one-way ANCOVA (controlling for age, sex, family history, disease duration, and multiple comparisons) exhibited no significant difference between these two groups in JME patients (Figure 3).

### 3.2. Correlation Analysis

Partial correlation analysis controlling for age, sex, family history, and multiple comparisons in patients with JME resulted in no significant associations between disease duration and FAB scores (Table 5).

We further conducted partial correlation analysis in two subgroups of with or without recent (within less or more than a week) seizure (Table 6). In JME patients with recent seizure, disease duration was correlated with conceptualization, mental flexibility, inhibitory control, and total FAB score (Pearson correlation coefficient = 0.958, 0.840, 0.823, and 0.950 and FDR < 0.001, <0.05, <0.05, and <0.001, respectively). In contrast, we found no significant association in JME patients without recent seizures.

### 3.3. Diagnostic Efficiency Analysis

**Figure 4:**
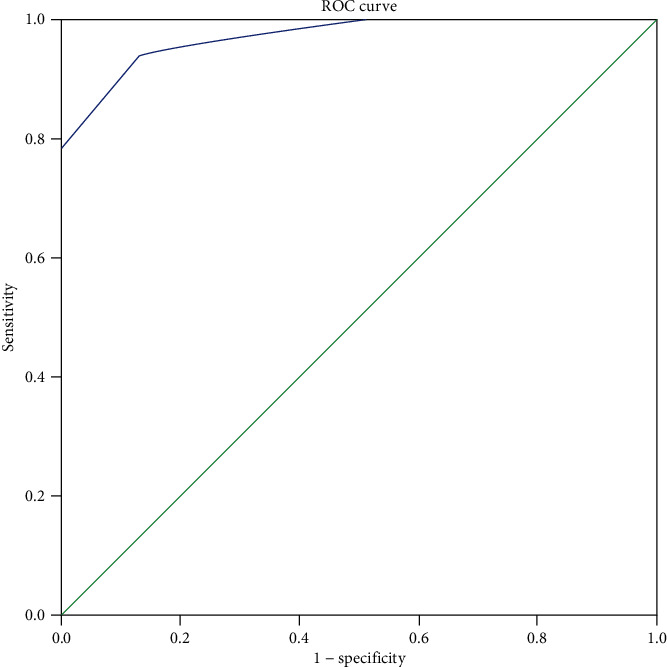
ROC curve discriminating between JME patients and HCs for FAB total score.

Finally, we used the ROC analysis to examine the value of FAB in discriminating JME patients with HCs and determine the optimal cutoff value for FAB total and subset scores. The value of the AUC was 0.971 (95% confidence interval (CI): 0.947–0.994) for FAB total score (Figure 4). The ideal cutoff for FAB total score was 15.5, rendering the maximum sensitivity and specificity (Table 7).

**Figure 5:**
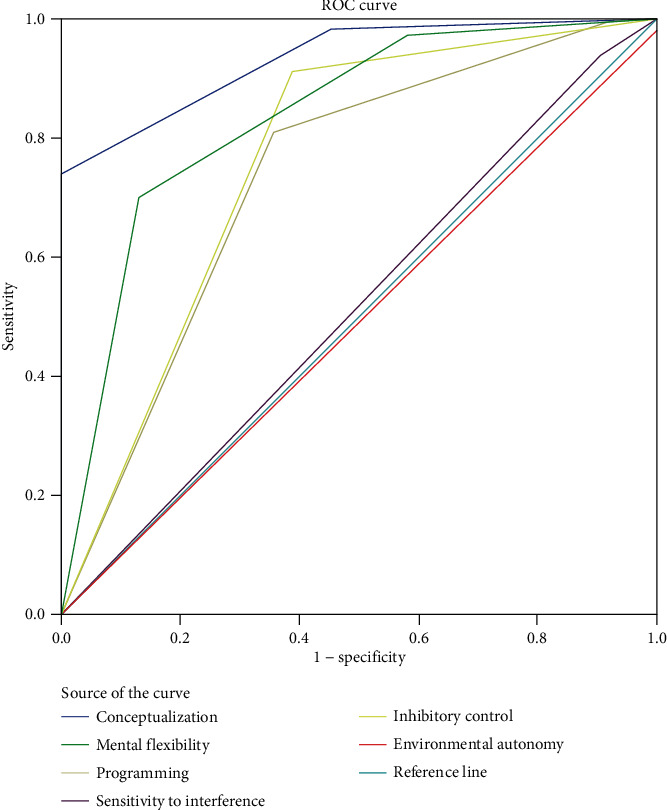
ROC curve discriminating between JME patients and HCs for FAB subset scores.

The diagnostic value of FAB subsets is depicted in Figure 5. We found strong discriminant capacity in conceptualization (AUC = 0.933; 95% CI: 0.973-894), mental flexibility (AUC = 0.836; 95% CI: 0.921-751), fair discriminant capacity in inhibitory control (AUC = 0.762; 95% CI: 0.872-653), programming (AUC = 0.733; 95% CI: 0.842-625), poor/failed discriminant capacity in sensitivity to interference (AUC = 0.517; 95% CI: 0.634-399), and environmental autonomy (AUC = 0.491; 95% CI: 0.605-377).

## 4. Discussion

In this study, executive functioning in JME patients was assessed using an easy-to-administer neuropsychological measure, known as FAB. The major findings of this study are as follows: (1) comparing with HCs, JME patients showed lower total FAB score and lower scores in most domains consisting of conceptualization, mental flexibility, programming, and sensitivity to interference (all domains except inhibitory control and environmental autonomy); (2) disease duration was associated with conceptualization, mental flexibility, inhibitory control, and total FAB score only in JME patients with a recent seizure; (3) duration of the time since the last seizure had no significant effect on JME patients' FAB scores; (4) the family history of epilepsy showed no significant effect on FAB scores in JME patients; (5) the number of AEDs (polytherapy versus monotherapy) had no significant effect on FAB scores in JME patients; and (6) we found that FAB total score and conceptualization and mental flexibility scores can efficiently discriminate between JME patients and HCs.

We, here, conducted a comprehensive review on the current literature of JME-related executive deficits in order to compare our results from FAB as a bedside screening tool with the results of extended neuropsychological batteries. The majority of reviewed studies have used digit span, TMT, Stroop test, and verbal fluency to investigate the executive deficits in JME patients. Valente et al. [20] and Cevik et al. [21] showed lower performance in digit forward and backward test in JME patients compared to HCs. However, Abarrategui et al. [22] found no difference. Digit span was also shown to be correlated with clinical variables, such as age, education, and duration seizures [21]. Digit backward test involves the manipulation of numbers and requires intact executive functioning. Almost all studies reported worse performance on either TMT A or B in patients with JME [9, 20, 21, 23–25]. TMT A is a test of visual attention, while TMT B is accompanied by task switching and thus involves inhibitory control and mental flexibility. TMT scores were associated with age, age of onset, disease duration, and drug load [21, 23]. The majority of studies demonstrated that JME is associated with worse performance in different subsets of Stroop or color-word interference tests [9, 19–23, 25], which were associated with clinical variables [19, 21, 23]. Stroop subtests assess different domains of executive functions, including inhibition and switching, sensitivity to interference, and mental flexibility. In addition, three studies reported deficits in fluency tests, including letter [9], semantic and phonemic [24], and figure [25] fluency. Ultimately, Cevik et al. [21] substantiated that patients with JME have lower scores in Abstraction and Judgment tests, implying impaired conceptualization in JME. In comparison with the results of these comprehensive neuropsychological assessments, our findings from FAB demonstrated that JME is associated with deficits in conceptualization, mental flexibility, programming, and sensitivity to interference but not in inhibitory control and environmental autonomy. Deficits in conceptualization, mental flexibility, and inhibitory control were correlated significantly with disease duration but not with the number of AEDs.

Furthermore, our diagnostic efficiency analysis demonstrated that FAB total or subset scores could be used as reliable and supplementary bedside tools for the diagnosis of JME. In this regard, FAB total score along with conceptualization and mental flexibility scores showed the highest diagnostic capacity. In line with our findings, previous studies have shown that FAB is also a sensitive discriminant tool in other neurological disorders, including obstructive sleep apnea [26], frontotemporal lobar degeneration [27], and stroke [28].

Although it seems that FAB is a more sensitive test for evaluating the frontal lobe function, which has brought about some clinical indications for it in diseases with frontal lobe impairments [29–31], some studies have shown the involvement of other brain regions in completing the task. However, lower FAB scores might more accurately refer to a frontal lobe dysfunction in patients with JME than previously used tests for assessing executive function. In fact, it was shown that FAB scores could be explained by the scores of other neuropsychological tests such as Word List Memory, Constructional Praxis, Constructional, Recall, and Stroop color-word test. The performance on these tests is dependent on both frontal and nonfrontal regions, including the precuneus, right temporoparietal region, and posterior cingulate cortex. Build upon this, we found that JME patients have deficits in various subsets of FAB, which might not be just the result of frontal dysfunctioning but also because of the disruptions in the connections between frontal regions with other nonfrontal regions.

In a previous study on TLE patients, the authors reported that patients with a recent seizure achieved lower scores in mental flexibility domain and total FAB. Animal and human FDG-positron emission tomography studies also demonstrated that duration of time elapsed since the last seizure is in a negative association with brain metabolism [32–34]. Our study is the first one to address this issue in JME patients. Nonetheless, we did not observe such a relationship in JME patients as none of the FAB total or domain scores were significantly different between patients with or without a seizure within the last week. The reason behind this inconsistency may be that these two epilepsy syndromes have different involvement of brain structures [35, 36].

Similar to our study, several previous studies have evaluated the association between cognitive deficits of JME patients and different clinical variables. Previous results are not entirely consistent. Duration of epilepsy was exhibited to have a significant effect on the cognitive performance of patients with JME [6, 37]. In our study, disease duration was associated with conceptualization, mental flexibility, inhibitory control, and total FAB score only in JME patients with a recent seizure but not in the JME group with a distant seizure, which indicates the importance of time proximity of seizure on the executive functions in JME patients. Observation of cognitive impairments, including executive dysfunction in siblings of patients with JME, has proved the potential value of genetic factors in determining the severity of executive dysfunction in JME [38]. Regarding the family history, one study [6] observed no association, while another study by Pascalicchio et al. [39] reported an association between family history and cognitive functions. In the study by Sonmez et al. [10], a more widespread cognitive impairment and a frontal lobe dysfunction were observed in patients with a positive family history. Notwithstanding, in this study, we did not observe any differences between patients with or without a family history.

Eventually, although FAB is an appropriate test for detection of executive deficits in different neurological and psychiatric disorders, it should be noted that it is not a comprehensive test and is not designed to cover all aspects of cognitive functions. Instead, FAB is a brief and feasible bedside measure assisting the clinicians in screening for early frontal-dependent cognitive dysfunctions in patients with neurological disorders like JME. In contrast to other neuropsychological tests, which are highly time-consuming, the succinctness of FAB has made it a handy and feasible measure, which is the reason why it could be widely applied in clinical settings in the future. More comprehensive neuropsychological tests might be administered after FAB in order to determine the exactly affected domains of cognitive functions.

The main limitations of this study were as follows: (1) The source of information for dividing our patients to “less than a week” and “a week or more” subgroups was patient and/or his/her family reports, and we could not double-confirm this with an alternative source. (2) The severity, frequency, and type of seizures were not documented in this study. (3) We did not provide exact information on the level of education in our patients and HCs. (4) The type of AED treatment and age of onset are not documented. (5) FAB is a battery of the bedside type and has been created in clinical and age contexts different from what we used in this study.

## 5. Conclusion

Collectively, we demonstrated that patients with JME have deficits in some aspects of frontal-mediated executive functions compared to HCs. FAB is an appropriate clinical tool for evaluation of executive functions in these patients, as it is an available test for identifying frontal-dependent executive deficits. The time elapsed since the last seizure may not be a precise predictor for executive functioning in JME patients. Our study is the first one investigating the FAB as a feasible in-clinic tool in patients with JME and its association with clinical and demographic characteristics of JME. However, given the limitations above, further studies are needed to compare this tool to other neuropsychological measures in JME patients.

## Figures and Tables

**Table 1:** Demographic and clinical characteristics of patients and healthy controls.

	(A) JME patients (*n* = 31)	(B) Healthy controls (*n* = 110)	*P* value
Age (mean ± SD)^a^	22.48 ± 6.23	27.80 ± 9.93	0.005
Sex (male/female)^b^	5/26	24/86	0.489
Disease duration (mean ± SD)^a^	10.53 ± 6.15	—	—
Family history (*n*, %)^b^	14 (45.2%)	4 (3.6%)	<0.00001
GTCS (*n*, %)^b^	26 (83.9%)	—	—
Time from the last seizure (*n*, %)^b^		—	—
Within 1 week	9 (29%)		
A week or more	22 (71%)		
Treatment (*n*, %)^b^		—	—
Monotherapy	18 (58.1%)		
Polytherapy	13 (41.9%)		
Total FAB score^a^	13.38 ± 1.81	17.01 ± 0.96	<0.00001

^a^Student *t*-test. ^b^Chi-square. JME: juvenile myoclonic epilepsy; GTCS: generalized tonic-clonic seizure; FAB: Frontal Assessment Battery; SD: standard deviation.

**Table 2:** Comparing FAB scores between patients and healthy controls controlling for age, sex, family history, disease duration, time from the last seizure, and multiple comparison.

	(A) JME patients (*n* = 31)	(B) Healthy controls (*n* = 110)	FDR (Cohen's *d*)
Conceptualization (mean ± SD)	1.25 ± 0.77	2.71 ± 0.49	<0.00001 (2.26)
Mental flexibility (mean ± SD)	1.61 ± 0.84	2.66 ± 0.56	<0.00001 (1.60)
Programming (mean ± SD)	2.29 ± 0.58	2.80 ± 0.39	<0.00001 (1.03)
Sensitivity to interference (mean ± SD)	2.90 ± 0.30	2.93 ± 0.24	<0.00001 (0.11)
Inhibitory control (mean ± SD)	2.32 ± 0.65	2.90 ± 0.28	0.608
Environmental autonomy (mean ± SD)	3.00 ± 0.00	2.97 ± 0.21	0.612

JME: juvenile myoclonic epilepsy; FDR: false discovery rate; SD: standard deviation.

**Table 3:** Comparing FAB scores within JME patients controlling for age, sex, family history, disease duration, and multiple comparison.

JME	Less than a week (*n* = 9)	A week or more (*n* = 22)	FDR
Conceptualization (mean ± SD)	1.11 ± 0.78	1.31 ± 0.77	0.635
Mental flexibility (mean ± SD)	1.33 ± 0.86	1.72 ± 0.82	0.597
Programming (mean ± SD)	2.44 ± 0.72	2.22 ± 0.52	0.597
Sensitivity to interference (mean ± SD)	3.00	2.86 ± 0.35	0.597
Inhibitory control (mean ± SD)	2.44 ± 0.52	2.27 ± 0.70	0.635
Environmental autonomy (mean ± SD)	3.00	3.00	1
Total FAB score (mean ± SD)	13.33 ± 1.93	13.40 ± 1.81	1

JME: juvenile myoclonic epilepsy; FAB: Frontal Assessment Battery; FDR: false discovery rate; SD: standard deviation.

**Table 4:** Comparing FAB scores between monotherapy and polytherapy groups controlling for age, sex, family history, disease duration, and multiple comparison.

JME	Monotherapy (*n* = 18)	Polytherapy (*n* = 13)	FDR
Conceptualization (mean ± SD)	0.94 ± 0.63	1.69 ± 0.75	0.063
Mental flexibility (mean ± SD)	1.33 ± 0.84	2.00 ± 0.70	0.318
Programming (mean ± SD)	2.33 ± 0.68	2.23 ± 0.43	0.898
Sensitivity to interference (mean ± SD)	2.88 ± 0.32	2.92 ± 0.27	0.900
Inhibitory control (mean ± SD)	2.11 ± 0.67	2.61 ± 0.50	0.273
Environmental autonomy (mean ± SD)	3.00	3.00	1
Total FAB score (mean ± SD)	12.61 ± 1.85	14.46 ± 1.12	0.063

JME: juvenile myoclonic epilepsy; FAB: Frontal Assessment Battery; FDR: false discovery rate; SD: standard deviation.

**Table 5:** Partial correlation between disease duration and FAB scores in JME patients controlling for age, sex, family history, and multiple comparison.

		Conceptualization	Mental flexibility	Programming	Sensitivity to interference	Inhibitory control	Environmental autonomy	Total FAB
JME	Disease duration	0.235	0.183	-0.052	-0.114	0.145	-	0.197

-The presented values are Pearson correlation coefficients. No significant correlation was found. JME: juvenile myoclonic epilepsy; FAB: Frontal Assessment Battery.

**Table 6:** Partial correlation between disease duration and FAB scores in “less than a week” and “a week or more” groups of JME patients, controlling for age, sex, and multiple comparison.

		Conceptualization	Mental flexibility	Programming	Sensitivity to interference	Inhibitory control	Environmental autonomy	Total FAB
Less than a week	Disease duration	0.967∗∗	**0.813**	0.454	-	**0.784**	-	0.967∗∗
A week or more	Disease duration	-0.005	-0.058	-0.012	-0.012	-0.68	-	-0.004

-The presented values are Pearson correlation coefficients. JME: juvenile myoclonic epilepsy; FAB: Frontal Assessment Battery. ∗∗FDR < 0.001. ∗FDR < 0.01. Bold: FDR < 0.05.

**Table 7:** ROC cutoff points for FAB total scores.

Positive if greater than	Sensitivity	Specificity
7	1	1
9.5	1	0.968
11.5	1	0.839
12.5	1	0.742
13.5	1	0.516
**14.5**	**0.964**	**0.258**
15.5	0.936	0.129
16.5	0.782	<0.0001
17.5	0.336	<0.0001
19	<0.0001	<0.0001

Bold: cutoff point maximizing the sum of sensitivity and specificity.

## Data Availability

The data that support the findings of this study are available from the corresponding author, upon reasonable request.
